# Composite measures of quality of health care: Evidence mapping of methodology and reporting

**DOI:** 10.1371/journal.pone.0268320

**Published:** 2022-05-12

**Authors:** Pinar Kara, Jan Brink Valentin, Jan Mainz, Søren Paaske Johnsen

**Affiliations:** 1 Danish Center for Clinical Health Services Research (DACS), Department of Clinical Medicine, Aalborg University, Aalborg, Denmark; 2 Psychiatry, Aalborg University Hospital, Aalborg, Denmark; 3 Department for Community Mental Health, University of Haifa, Haifa, Israel; 4 Department of Health Economics, University of Southern Denmark, Odense, Denmark; University of Malta Faculty of Health Sciences, MALTA

## Abstract

**Background:**

Quality indicators are used to quantify the quality of care. A large number of quality indicators makes assessment of overall quality difficult, time consuming and impractical. There is consequently an increasing interest for composite measures based on a combination of multiple indicators.

**Objective:**

To examine the use of different approaches to construct composite measures of quality of care and to assess the use of methodological considerations and justifications.

**Methods:**

We conducted a literature search on PubMed and EMBASE databases (latest update 1 December 2020). For each publication, we extracted information on the weighting and aggregation methodology that had been used to construct composite indicator(s).

**Results:**

A total of 2711 publications were identified of which 145 were included after a screening process. Opportunity scoring with equal weights was the most used approach (86/145, 59%) followed by all-or-none scoring (48/145, 33%). Other approaches regarding aggregation or weighting of individual indicators were used in 32 publications (22%). The rationale for selecting a specific type of composite measure was reported in 36 publications (25%), whereas 22 papers (15%) addressed limitations regarding the composite measure.

**Conclusion:**

Opportunity scoring and all-or-none scoring are the most frequently used approaches when constructing composite measures of quality of care. The attention towards the rationale and limitations of the composite measures appears low.

**Discussion:**

Considering the widespread use and the potential implications for decision-making of composite measures, a high level of transparency regarding the construction process of the composite and the functionality of the measures is crucial.

## Introduction

Quality of health care is essential for all healthcare stakeholders, including, patients, healthcare providers, institutes, insurers, policy makers, and government. Hence, valid assessment of the quality is crucial in order to monitor, evaluate and improve the quality of healthcare services. Quality indicators are measurement tools that are used to quantify the quality of care. They serve various purposes such as documenting the quality, benchmarking, setting priorities, facilitating quality improvement and supporting patient choice of providers. The indicators can be classified in various ways with Donabedian’s structure, process and outcome indicator classification being the most frequently adopted approach [1]. The use of quality indicators is widespread in health care and the number of indicators is huge. Consequently, there is an increasing interest for composite measures based on a combination of multiple indicators.

A composite measure can be defined as a combination of multiple individual indicators [2]. Individual indicators are useful for measuring specific aspects of quality, however, an overall measure that reflects multiple aspects and dimensions of quality may have considerable advantages over individual indicators. Indeed, composite indicators can summarize the quality of care as a single value. Hence, they can be helpful when comparing, rating, ranking and selecting healthcare providers as an alternative to assessing providers’ performance according to many individual indicators [3]. Using composite indicators rather than individual indicators may also result in increased reliability since the combination of multiple individual indicators will imply that the underlying number of observations is larger [4].

However, composite indicators also come with limitations. Differences and relationships between individual indicators may be masked and information regarding specific aspect(s) of performance can be lost [2]. Using different approaches for construction of the composite measures may give different results. In other words, composite measures can be sensitive to the methodology that has been used [5]. Therefore, if the construction process for the composite indicators is not transparent, composite indicators may be misused and if not constructed in a methodologically sound way, the results obtained by using these indicators (such as hospital rankings) may not be reliable [6].

Detailed recommendations on the construction of reliable composite indicators have been published previously [2, 7]. The steps include development of a theoretical framework, selection of indicators, multivariable assessment of indicators, weighting and aggregation of indicators and as the last step, validation of the composite indicator [2, 7].

The aim of this study was to investigate the use of composite measures of quality of care based on process indicators in the peer-reviewed literature. In addition, we examined whether methodological considerations were provided in the publications.

## Methods

This review was done in accordance with the recommendations in the PRISMA statement [8].

### Definitions and terminology

Different approaches for construction of composite indicators exist, which mostly differ in terms of weighting and aggregation of individual indicators. Some of the methods to construct composite indicators are introduced (Table 1).

**Table 1:** Examples of methods for constructing composite measures.

Methods	Definition
**Overall percentage (Opportunity scoring)**	The composite score is calculated as the total number of processes of care delivered to all patients divided by the total number of eligible care processes [9]. $\text{Overall}\;\text{percentage}=\frac{\sum_{\text{i}=1}^{\text{n}}\text{Number}\;\text{of}\;\text{processes}\;\text{provided}\;\text{to}\;\text{patient}\;\text{i}}{\sum_{\text{i}=1}^{\text{n}}\text{Number}\;\text{of}\;\text{processes}\;\text{patient}\;\text{i}\;\text{is}\;\text{eligible}\;\text{for}}$ where n denotes the number of patients in a provider (or other level of interest).
**Patient average (Opportunity scoring)**	Composite scores are calculated for each patient (number of care processes delivered divided by number of patient specific eligible care processes) and can then be averaged to obtain provider-level composite scores [9]. $\text{Score}\;\text{for}\;\text{patient}\;\text{i}=\frac{\text{Number}\;\text{of}\;\text{processes}\;\text{provided}\;\text{to}\;\text{patient}\;\text{i}}{\text{Number}\;\text{of}\;\text{processes}\;\text{patient}\;\text{i}\;\text{is}\;\text{eligible}\;\text{for}}$ $\text{Composite}\;\text{score}\;\text{for}\;\text{provider}=\frac{\sum_{\text{i}=1}^{\text{n}}\text{Score}\;\text{for}\;\text{patient}\;\text{i}}{\text{n}}$ where n denotes the number of patients in a provider (or other level of interest).
**Indicator average**	For each indicator the percentage of times that indicator is fulfilled is calculated and then averaged across all indicators [9]. $\text{Score}\;\text{for}\;\text{indicator}\;\text{j}=\frac{\text{Number}\;\text{of}\;\text{times}\;\text{indicator}\;\text{j}\;\text{is}\;\text{provided}}{\text{Number}\;\text{of}\;\text{times}\;\text{patients}\;\text{were}\;\text{eligible}\;\text{for}\;\text{indicator}\;\text{j}}$ $\text{Composite}\;\text{score}\;\text{for}\;\text{provider}=\frac{\sum_{\text{j}=1}^{k}\text{Score}\;\text{for}\;\text{indicator}\;\text{j}}{\text{k}}$ where k denotes the number of indicators.
**All-or-none (defect-free scoring)**	Composite measure is calculated on patient level. Each patient gets either 1 (all eligible care processes are fulfilled) or 0 (at least 1 of the eligible care processes is unachieved). This approach can be preferred especially (1) when process indicators interact or partial achievement of a series of steps is insufficient to obtain the desired result, (2) when adherence rates for indicators are very high so using methods that award partially provided care will neither be helpful in order to distinguish between providers’ performance nor motivates providers to improve the quality of care [10]. $\text{Score}\;\text{for}\;\text{patient}\;\text{i}=\{\begin{array}{c} 1,\;\text{All}\;\text{eligible}\;\text{processes}\;\text{are}\;\text{delivered}\;\text{to}\;\text{the}\;\text{patient}\\ 0,\;\text{At}\;\text{least}\;\text{one}\;\text{eligible}\;\text{care}\;\text{process}\;\text{is}\;\text{not}\;\text{delivered} \end{array}$ $\text{Composite}\;\text{score}\;\text{for}\;\text{provider}=\frac{\sum_{\text{i}=1}^{\text{n}}\text{Score}\;\text{for}\;\text{patient}\;\text{i}}{\text{n}}$ where n denotes the number of patients in a provider (or other level of interest).
**70% standard and other thresholds**	This approach is similar to all-or-none scoring but using a lower threshold than 100% [8].

In patient average, all-or-none scoring and 70% standard approaches, composite scores are calculated at patient-level and requires patient-level information. The scores obtained for each patient can subsequently be averaged to get provider-level, region-level or other levels of interest scores.

There are several approaches for assigning weights to individual indicators before aggregating them into composites. Some of the methods to assign weights to individual indicators are provided (Table 2).

**Table 2:** Examples of weighting approaches for constructing composite measures.

Weighting approach	Definition
**Equal weights**	All indicators receive the same weight. This approach generally indicates that all indicators are equally important in the composite [7].
**Expert weights**	An expert panel assigns weights to individual indicators depending on the panel’s criteria, such as indicators’ importance, impact, evidence score, feasibility and reliability.
**Regression weights**	Each indicator is weighted according to the degree of its association with an outcome, e.g., 30-day mortality. Using regression weights, the indicator with the strongest association with the outcome receives the highest weight [11]. This approach may be preferred if there is a gold standard end point.
**Principle component analysis-based weights**	PCA-based weights may be preferred when individual indicators are highly correlated. In this approach, correlated indicators are grouped, since they may share underlying characteristics. In this approach, each indicator is weighted according to its proportional factor loading [12]. This should not be confused with methods, in which factor analysis is used only as a part of the selection process for individual indicators.

Note that in the literature opportunity scoring is sometimes also referred to as *denominator-based weights approach* and is a weighted average for which the weights are the rate of eligibility for each indicator. In our review, we preferred to distinguish between this kind of weights which occurs naturally due to aggregation method and the weights which are additionally assigned to indicators by investigators according to each indicator’s association with the outcome, reliability, feasibility, importance or an expert judgement. Therefore, if opportunity scoring is used in a study without further assignment of weights to individual indicators and no differentiation is made between indicators, we referred this method as “opportunity scoring with equal weights”. Furthermore, the scores obtained by using patient average, overall percentage and indicator average methods will be the same if all of the patients are eligible for all indicators, even though the interpretation of the results will differ. Finally, it should be emphasised that weighting individual indicators before aggregation is not relevant in all-or-none scoring.

### Search strategy

We conducted a literature search to identify publications that used composite measures to assess quality of care. We queried the PubMed and EMBASE databases (latest update 1 December 2020), using the following terms: composite measures, quality of health care and other variations (S1 Appendix). We did not use any restrictions on date of publication in our search. The full search string is provided (S1 Appendix).

### Eligibility criteria

In this review, we included studies using composite measures based exclusively on process of care indicators. Process indicators (for example, β-blocker prescription at discharge for patients with acute myocardial infarction, oxygenation assessment for patients with pneumonia or eye examination for patients with diabetes) have some advantages over outcome indicators. First, these indicators reflect actual care delivered to the patients, hence, they can be more actionable. Second, outcome indicators like 30-day mortality or readmission rate may be influenced by confounding factors, e.g., age, sex, severity of underlying disease or level of comorbidity, which may not be completely eliminated by risk adjustment [13]. Third, process indicators are a particular appealing alternative in clinical scenarios where the most relevant outcome requires long follow-up time, e.g., recurrence of cancer [13]. Fourth, composite measures consisting of a combination of both process and outcome indicators comes with additional challenges due to the inherent problem of meaningful weighting and aggregation of these two different types of indicators (for example, assessment of the relative importance of providing CT scan to patients compared to an outcome indicator such as mortality). As a conclusion, we restrict this review to only process indicators as would not be feasible to cover composite measures of multiple types of indicators satisfactory in a single paper. However, we recognize the value of outcome indicators and an assessment of composite measures of outcome indicators could be a relevant scope for a separate study.

Whereas clinical process indicators reflect actual delivered care, indicators reflecting utilization or access to care were not included as they reflect a complex result of organizational factors, patient preferences and patient compliance [14]. These indicators are therefore not under the full control of the healthcare system.

Even though patient-reported indicators may carry useful information, they typically lack details on the timeliness and appropriateness of individual clinical processes, which are crucial when evaluating the quality of care. Therefore, the studies that used these types of indicators in the composite measure were also excluded in the current review.

The exclusion criteria were as follows: (1) the composite measure included other types of indicators besides process indicators, (2) the composite measure included indicators related to access or utilization, (3) the composite measure included patient-reported indicators, (4) the scientific contribution was a protocol, trial design, purely methodological, letter, comment, editorial or a review, (5) the publication was not in English and (6) full text was not available.

### Study selection

Study selection was performed using Rayyan [15]. Records were screened independently by two reviewers (PK and JBV).

### Information retrieved from included studies

For each publication, we extracted information on the weighting and aggregation methodology that had been used to construct the composite indicator(s).

In addition, we registered justifications made for the selected methodology. We defined “justification” as the presence of any stated methodological argument for the methodology that had been used in the individual publications. We preferred not to use a very strict and detailed criterion to prevent subjective use and understanding of the term. We also obtained information on limitations and advantages of using composite indicators stated in the included publications. Finally, we examined whether publications using a single approach for construction of composite measures mentioned any alternative approaches.

We preferred to accept publications that only provided reference to these information (for example, the publication did not describe limitations regarding use of composite indicators itself but informed the reader about presence of limitations and included a reference for further information) as “provided information”. We recognize that especially when the main aim is not to assess the use of composite indicators directly, but rather to use composite indicators to support operational use (for example, a study that investigates effect of a programme participation and uses composite indicators as a tool to support decision making), providing detailed information about composite indicators may seem out of the scope of the publications. However, it is still important to provide the reader with some information about presence of potential limitations in relation to these indicators and the approaches used to construct them.

## Results

### Study selection

**Fig 1:**
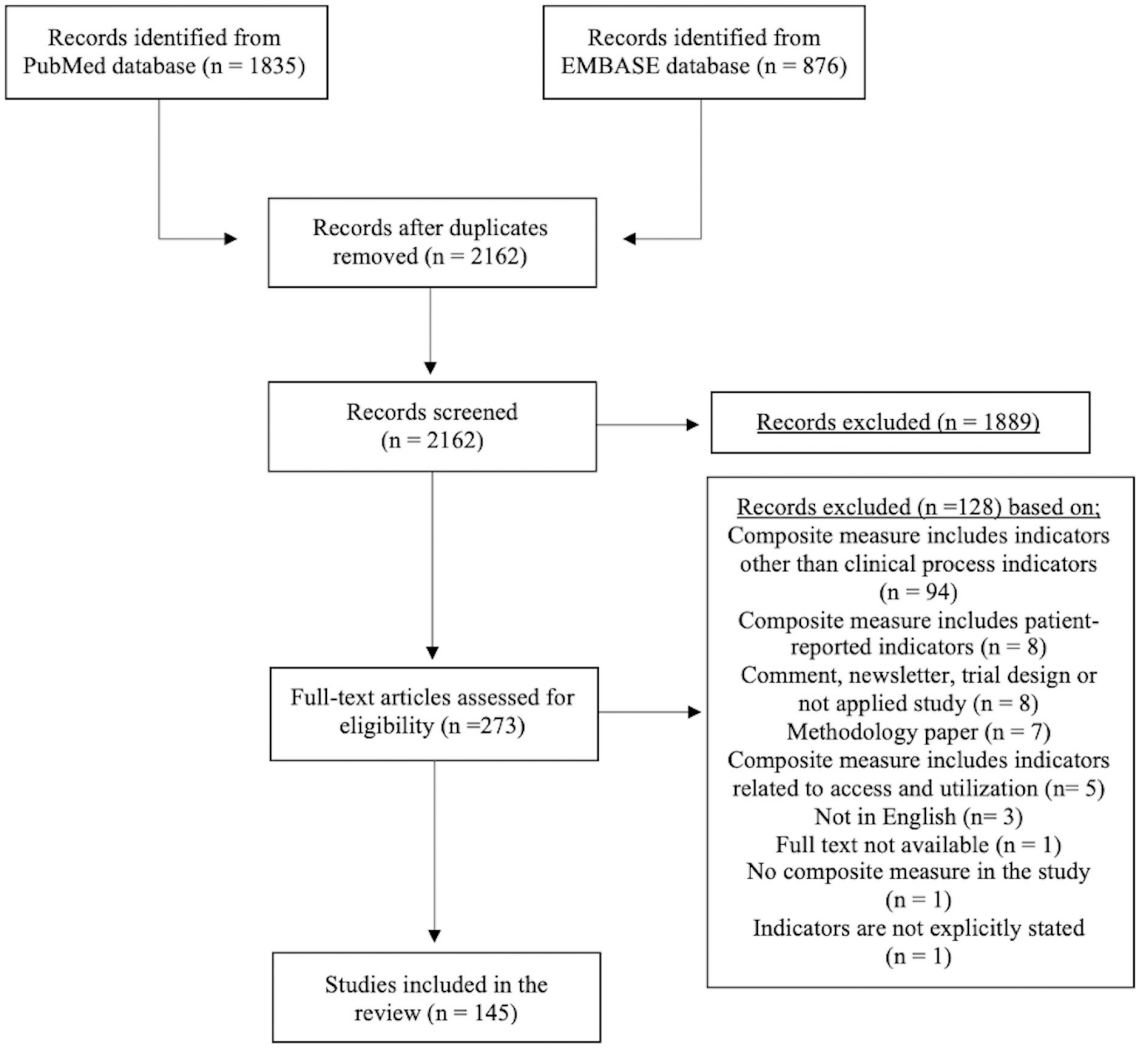
PRISMA diagram.

The search resulted in a total of 2711 publications, 1835 publications from PubMed database and 876 publications from EMBASE database. After removing 549 duplicates, 2162 unique publications were screened. First, title and abstracts were assessed for eligibility and 1889 publications were excluded since at least one of the exclusion reasons mentioned above was present. A total of 273 publications were included for the full-text screening and 145 of those met the full inclusion criteria. The list of included publications are provided (S1 Appendix). Detailed summary of the literature search is presented (Fig 1).

### Context

We categorized the publications according to (1) methodology that was used, (2) whether the publication used a single approach or multiple approaches to construct composite indicator(s), and (3) context. For context, we classified the primary objective of the publications under two main categories: operational use (for example, the composite indicator in the study was constructed to evaluate the effect of a quality improvement program or to compare performance of healthcare providers) and research purposes (for example, to investigate the association between process and outcome indicators or to assess the construction, use and implementation of composite indicators) (Table 3). We provided tables for characteristics of the included studies (S1 Table) and classification of context for each included publication (S2 Table).

**Table 3:** Examples for investigated questions in included publications.

Primary aim	Examples for investigated questions
**Operational use** (n = 61)	Effect of a program participation, implementation or intervention (n = 51) Is participation in Get with the Guidelines-Stroke program associated with improved quality of care? Does implementation of a clinical registry result in improved adherence to Stroke guidelines? Quality of care over time in a provider, Pure evaluation of quality of care in healthcare providers and/or comparison of healthcare providers (n = 10) Did quality of care improve over time for patients with AMI? How is adherence to standards of first-visit antenatal care among healthcare providers in Tanzania?
**Research purposes** (n = 84)	Association between process and outcome indicators (n = 25) Does adherence to process indicators lead to better outcomes? Is there an association between guideline concordance and risk of hospital admission? Association between hospital and/or patient characteristics and quality of care (n = 32) Are there disparities in the quality of health care across different socioeconomic groups? Are there age-dependent inequalities in quality of health care provided to patients? Is there an association between hospital volume and quality of care? Use, implementation or comparison of composite indicators (n = 26) Does composite indicator of quality discriminate hospital performance better than individual indicators? Can reliable and valid assessment of quality of care be achieved by creating composite indicators? Do hospital ranks change according to the method that has been used to construct composite indicators? Correlation between quality of care for heart failure and acute myocardial infarction (n = 1)

Of the publications classified as operational use (n = 61, 42%), 51 publications [16–66] investigated whether program participation or implementation of an intervention was associated with improved quality of care as measured by a composite indicator. In 10 publications [67–76] the primary aim was to measure hospital performance and/or changes in performance over time (Table 3).

Of the studies classified as research (n = 84, 58%), 25 publications [77–101] reported on the association between processes of care, assessed by one or more composite indicators, and outcome indicators. In 32 studies [102–133] the attention was on the link between hospital and/or patient characteristics and quality of care. Finally, other research aims were addressed in the remaining 26 studies [11, 12, 134–157] including whether composite indicators could better inform hospital performance than single indicators, the reliability and/or validity of composite indicators, development and implementation of composite indicators and the impact of using different methodologies for the construction of composite indicators. One study [158] investigated the correlation between quality of care for two clinical conditions (Table 3).

Out of 145 publications, three included composite measures for mental health care, including depression [145], bipolar disorder [146], and overall mental health care [94]. Two publications [52, 99] addressed both somatic and mental health care components, whereas the remaining 140 publications were focused on composite indicators for somatic diseases.

### Preferred composite score methodology

**Fig 2:**
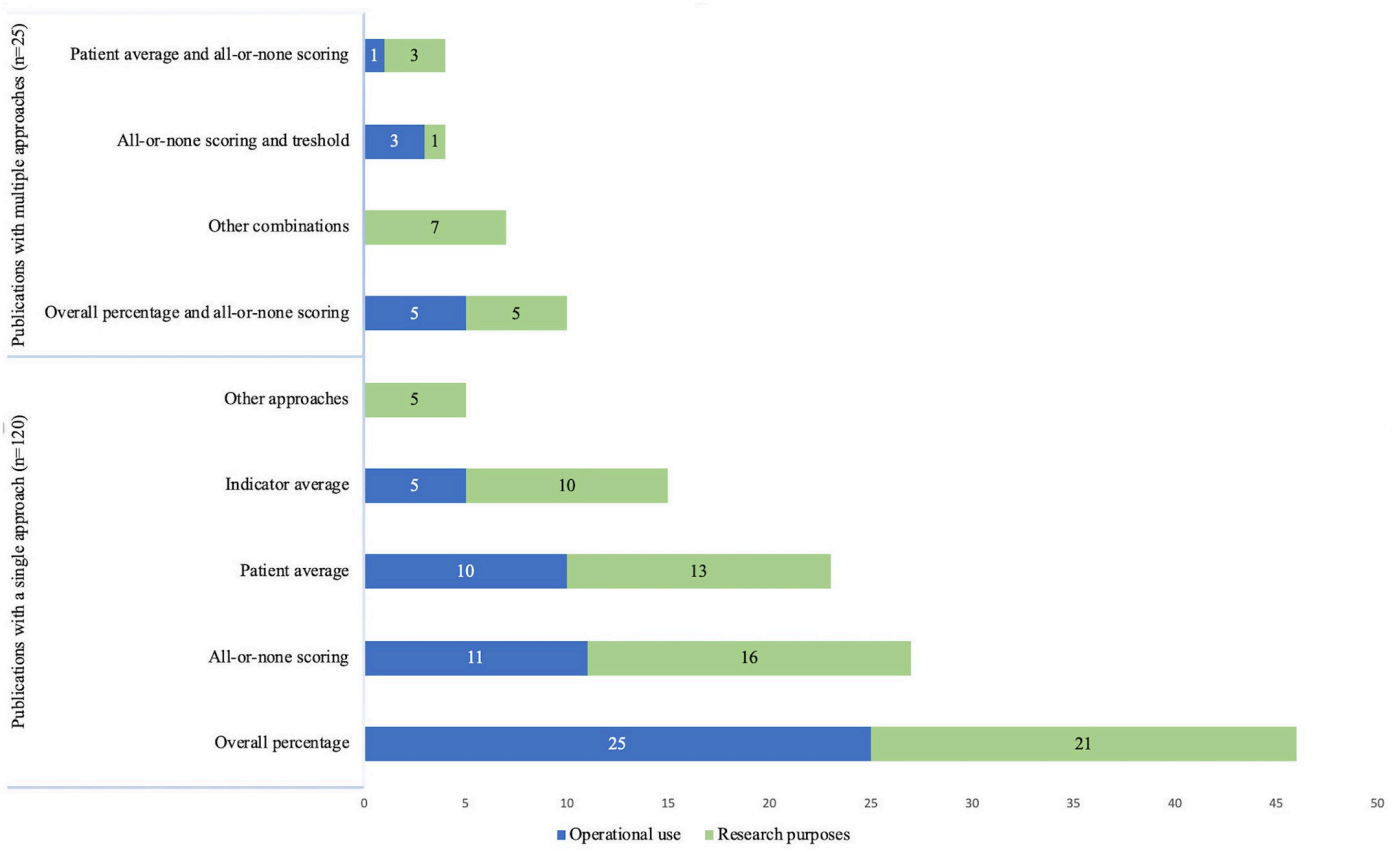
Preferred composite score methodology.

Opportunity scoring was the most used scoring method and represented in 89 (61%) publications. Of these, 62 (43%) publications had applied the overall percentage approach and 28 (19%) publications applied patient average. The second most used method was all-or-none scoring (n = 48, 36%). Out of 145 publications, 8 (6%) publications used 70% standard and other thresholds approach. Indicator average approach was present in 19 (13%) publications. Other approaches included two publications using latent variable models, one publication with principal component analysis and one publication with 70% standard approach on indicator level rather than patient-level (Fig 2). References corresponding to each aggregation method is provided (S3 Table). We also included a table to illustrate examples for selected process indicators for each methodology (S4 Table).

**Fig 3:**
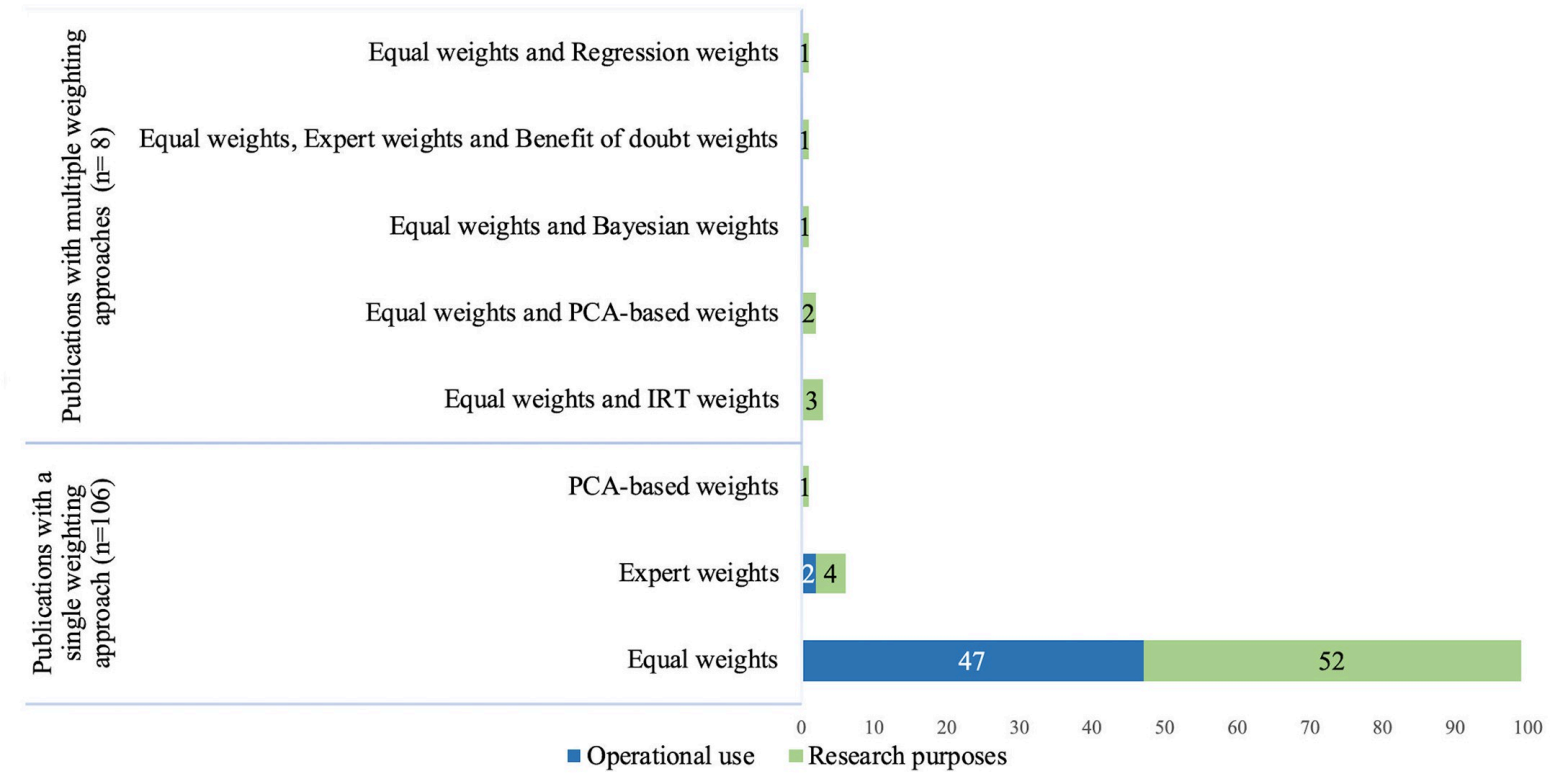
Weights used for constructing composite measures.

Weighting of individual indicators before aggregation were not relevant in the 27 publications using only all or none score approach. Of the remaining 118 publications, 107 used equal weights. Differential weights were present in 16 publications: 7 publications used weights obtained by expert opinion/subjective assessment, one publication used regression weights, three publications used weights obtained by item response theory, one publication used weights obtained by Bayesian approach, one publication used the benefit of doubt approach (assigning hospital specific weights in order to maximize performance) and three publications applied principal component analysis based weights (Fig 3). References corresponding to each weighting method is provided (S5 Table).

One publication [98] used patient specific weights according to care needs, i.e., care of patients is weighted by the natural logarithm of the total number of indicated care components, ln(n), to account for differences in number of needed care components for each case.

All of the studies that were conducted in order to support operational use preferred either equal weights or expert weights whereas more diverse approaches were used in publications with research purposes.

It was not possible to determine the methodology in four publications [22, 91, 95, 97]. In 26 publications, more than one aggregation and/or weighting approach were present.

### Other considerations and findings

Justification for the selected methodology used in the construction of the composite measure was found in 36 (25%) publications. The justifications included both limited justifications and justifications that were referenced (Table 4). Examples of the used justifications are provided (S6 Table). A summary of findings for each publication included in the review is provided (S2 Table).

**Table 4:** Methodological information in publications reporting composite measures of quality of health care based on process indicators.

Methodological information	Number of papers (%)	References
**Any justification for the selected methodology was provided or referenced**	36 (25%)	[11, 12, 23, 25, 27, 34, 54, 55, 57, 75, 86, 87, 98, 100, 118, 122, 123, 127, 134–136, 140–147, 149–153, 156, 157]
**Any limitation regarding composite measures is given or referenced**	22 (15%)	[11, 53, 75, 80, 98, 99, 116, 122, 134, 135, 139–142, 144–147, 149, 151, 152, 157]
**Any advantage regarding composite measures is given or referenced**	42 (29%)	[11, 23, 31, 38, 52, 53, 55, 59, 66, 75, 80, 83, 86, 87, 100, 101, 116, 122, 123, 127, 134–136, 138–156]
**For papers that used a single approach, presence of any other approach was mentioned or referenced**	10 (8%)	[75, 87, 93, 122, 123, 139, 142, 146, 152, 156]

Of 145 publications, methodological limitations of composite measures were addressed in 22 (15%) publications, including limitations that were referenced (Table 4). The reported limitations included concerns regarding loss of important information (n = 6, 4%), findings being sensitive to the choice of methodology for construction of the composite measures (n = 9, 6%), concerns over the construction process, such as weighting and aggregation methods or the selection of indicators included in the composite (n = 7, 5%), concerns over transparency (3, 2%) and oversimplifying complex data (n = 2, 1%).

Of 145 publications, 42 (29%) publications mentioned specific advantages of composite indicators (Table 4). Reported advantages included the comprehensiveness of the composite indicator (for example, summarizing overall quality, presents overall picture) (n = 26, 18%), facilitation of comparisons (n = 10, 7%), interpretability and being easier to understand (n = 8, 6%), increased reliability and stability (n = 6, 4%), and simplification (for example, reduced number of indicators and numbers in quality reports) (n = 5, 3%).

Of the 119 publications, which used a single composite score methodology, a total of 10 (8%) publications mentioned the presence of alternative methods for the construction of composite indicators (Table 4).

Some examples regarding methodological statements found in the literature is provided (S7 Table).

## Discussion

Despite the importance and widespread use of composite indicators to summarize the quality of health care, we found that methodological considerations were not addressed in the majority of the publications and that there was only modest variation regarding the chosen methodology for construction of the composite measure(s). Opportunity-based scoring, indicator average and all-or-none scoring were the most frequently preferred approaches to obtain composite measures, whereas use of other methods was sparse.

To our knowledge, this is the first review that investigates the use of composite measures of quality of health care based on process indicators. Some strengths of this review were: (1) It complied with the PRISMA guideline for systematic reviews whenever possible, (2) A large number of studies were included as we did not restrict our search to a specific clinical condition but considered all clinical conditions and disease areas relevant, (3) all publications were carefully screened by two independent reviewers to reduce possible bias.

This review has several limitations. First, it was restricted to peer-reviewed publications included in the PubMed and EMBASE databases. However, these two databases cover a substantial amount of publications within the field. Second, relevant publications might have been excluded if they were not in English. And third, although guidelines for validating composite measures of quality of health care have been developed [2], we did not investigate the extent to which the applied composite measures were validated in the publications under review, as this is a broad topic outside the scope of this review.

### Implications and recommendations

Composite measures have the advantage of summarizing the quality of care with a single number and have been increasingly used to evaluate the quality of healthcare services. One of the concerns regarding use of composite measures is the lack of a standard approach to construct them and possible effects and consequences of using different approaches. Several studies in the literature examined the effects of using different approaches regarding weighting and aggregation. However, the findings have been contradictory with some studies indicating that the use of different methods provided substantially different results, whereas others have found highly correlated results, e.g., in healthcare provider rankings, using different methods.

Jacobs et al. [5] investigated the effect of using different aggregation and weighting methods on hospital rankings and concluded that these measures are sensitive to the methodology and hospital rankings can change substantially depending on the methods that has been used. Simms et al. [11] constructed composite indicators for acute myocardial infarction care, using opportunity scoring with equal weights, opportunity scoring with regression weights and all-or-none scoring approaches. While these composite indicators were associated with the outcome, the rankings of hospitals were substantially influenced by the method that had been used to construct composite indicators.

In contrast, Eapen et al. [149] compared two methodologies that are most commonly used for composite indicators: overall percentage with equal weights and all-or-none scoring to examine their effects on hospital rankings for acute myocardial infarction care. In their study, the rankings obtained by these two methods were highly correlated (r = 0.93).

Kolfschoten et al. [150] investigated several types of composite measures both on patient-level (patient average, all-or-none and 70% standard) and hospital-level (overall percentage, indicator average, patient average, all-or-none and 70% standard) and these measures’ association with morbidity and mortality for patients with rectum carcinoma and colon carcinoma. They found that none of the patient-level composite measures were associated with the outcome, except for an association between the 70% standard method and morbidity for patients with rectum carcinoma. In contrast, all hospital-level composite indicators were associated with morbidity for both rectum carcinoma and colon carcinoma. This difference between patient level and hospital level composite measures was attributed to other factors that may have more effect on patient-level while on hospital-level, composite scores may better present the quality of care in a hospital. This finding indicates the importance of a clear framework for the composite measure and also the consideration regarding for what and by whom it will be used.

The requirements for specific clinical conditions should be taken into account when selecting the most suitable methodology. As an example, the two most commonly used approaches, opportunity-based scoring and all-or-none scoring, emphasize different aspects of quality. Opportunity-based scoring awards partial performance and improvements, whereas all-or-none scoring promotes excellence and defect-free care. While using all-or-none scoring can be more suitable for conditions that require 100% adherence and anything other than ideal care is not enough to achieve success, opportunity-based scoring can be more useful to investigate and award improvements over time. For statistical approaches for construction, using principal component analysis can be beneficial when individual indicators are highly correlated with each other and can be grouped together (for example, a composite indicator with multiple care dimensions for diagnosis, treatment and consultation), whereas regression weights can be considered when there is a gold standard end point (for example, mortality).

Selection of individual indictors to be included in the composite requires careful evaluation. Investigating overall structure of the dataset including correlations and interrelationships between indicators may be useful and important to have meaningful composite measures and to prevent possible problems, such as double counting and can be a primer for the decision regarding assigning weights to individual indicators. Including clinical experts (for example, by establishing an expert panel) for indicator selection and weighting of indicators can be also considered in order to achieve potentially more clinically meaningful composite indicators.

Validation of the final composite indicator is an important step of construction in order to assure the composite indicator is fit for purpose, reliable, accurate and robust. National Quality Forum states that even if studies use already validated individual indicators, the final composite measure may not be the true reflection of quality after weighting and aggregation steps [159]. Hence, a separate validation process of the composite is still warranted in order to obtain a reliable composite score. Although it may be difficult to select the most suitable methodology to construct composite measures and perform validation when a study lacks gold standard, readers should be informed about possible limitations and challenges regarding the specific composite measures and the presence of alternative approaches.

## Conclusion

This review provides an overview of the methodologies for composite measures used in the peer-reviewed literature to evaluate the quality of care based on process indicators, including the justifications and methodological considerations made regarding these measures. An increased awareness among researchers and healthcare professionals is warranted regarding the presence of alternative methodologies and the importance of a transparent and robust methodology when constructing and reporting composite measures of process quality of health care.

## Supporting information

S1 Appendix Search strategy. (DOCX)

S1 Checklist PRISMA checklist. (DOC)

S1 Table Characteristics of included publications. (DOCX)

S2 Table Context, methods and methodological considerations in included publications. (DOCX)

S3 Table Aggregation methods used in publications. (DOCX)

S4 Table Examples for included indicators for each aggregation method. (DOCX)

S5 Table Weighting methods used in publications. (DOCX)

S6 Table Justifications for selected methodologies as stated in the included publications. (DOCX)

S7 Table Examples for methodological considerations counted as “provided”. (DOCX)
