# Supplementary material for: Composite measures of quality of health care: Evidence mapping of methodology and reporting
Source: PLoS One. 2022 May 12;17(5):e0268320. doi: 10.1371/journal.pone.0268320 (PMC9098058; doi:10.1371/journal.pone.0268320)
Supplement: S1 Appendix — (DOCX) [file pone.0268320.s001.docx]

**S1 Appendix.** Search strategy

Search for PubMed database (latest update 1 December 2020)

1. Composite measure* [tw]
2. Composite score* [tw]
3. Composite indicator* [tw]
4. Composite index* [tw]
5. 1 or 2 or 3 or 4
6. Quality of healthcare [tw]
7. Quality of health care [tw]
8. Healthcare performance [tw]
9. Health care performance [tw]
10. Healthcare qualit* [tw]
11. Health care qualit* [tw]
12. Health provider qualit* [tw]
13. Quality of health care [Majr]
14. 6 or 7 or 8 or 9 or 10 or 11 or 12 or 13
15. 5 and 14

Search for EMBASE database (latest update 1 December 2020)

1. Composite measure*:ti,ab,kw
2. Composite score*:ti,ab,kw
3. Composite indicator*:ti,ab,kw
4. Composite index*:ti,ab,kw
5. 1 or 2 or 3 or 4
6. Quality of healthcare*:ti,ab,kw
7. Quality of health care*:ti,ab,kw
8. Quality of care*:ti,ab,kw
9. Healthcare qualit*:ti,ab,kw
10. Health care qualit*':ti,ab,kw
11. Health provider qualit*:ti,ab,kw
12. Healthcare performance*:ti,ab,kw
13. Health care performance*:ti,ab,kw
14. Health care quality/exp/mj
15. 6 or 7 or 8 or 9 or 10 or 11 or 12 or 13 or 14
16. 5 and 15
17. 15 not 'conference abstract'/it
