# Supplementary material for: Composite measures of quality of health care: Evidence mapping of methodology and reporting
Source: PLoS One. 2022 May 12;17(5):e0268320. doi: 10.1371/journal.pone.0268320 (PMC9098058; doi:10.1371/journal.pone.0268320)
Supplement: S1 Table — (DOCX) [file pone.0268320.s003.docx]

**S1 Table. Characteristics of included publications**

| Publication | Year | Country | Clinical condition  care/procedure type | Primary objective(s) of the study |
| --- | --- | --- | --- | --- |
| Min et al. [107] | 2005 | USA | Overall | To investigate the factors associated with quality of care provided for vulnerable older patients. |
| Correa-de-Araujo et al. [109] | 2006 | USA | Diabetes | To investigate gender differences across racial and ethnic groups in the quality of care for diabetes. |
| Halterman et al. [135] | 2006 | USA | Asthma | To develop a composite index to measure and quantify preventive asthma care delivered in the primary care office. |
| Landon et al. [111] | 2006 | USA | AMI, HF, PN | To investigate hospital-level factors associated with quality of care for the treatment of AMI in US hospitals. |
| Lindenauer et al. [110] | 2006 | USA | COPD | To evaluate the quality of care provided to patients hospitalized for acute exacerbations of COPD and to determine whether hospital or patient characteristics influence treatment. |
| Williams et al. [75] | 2006 | USA | AMI, HF | To evaluated how well hospitals ranked on the US News & World Report list of heart and heart surgery hospitals performed on AMI and HF measures derived from ACC and AHA clinical treatment guidelines. |
| Glickman et al. [24] | 2007 | USA | AMI | To determine if pay for performance was associated with improved processes of care and outcomes for AMI at hospitals participating in the CMS pilot project. |
| Halasyamani et al. [137] | 2007 | USA | AMI, PN, HF | To compare the ranking of U.S. News and World Report's "Best Hospitals" with Hospital Compare performance ratings. |
| Krantz et al. [25] | 2007 | USA | CAD | To assess evidence-based medication use in CAD patients after initiation of a quality-improvement program at Denver Health. |
| Landon et al. [26] | 2007 | USA | Asthma, Diabetes, Hypertension | To investigate whether participation in the Health Disparities Collaboratives significantly improved the quality of care. |
| Lindenauer et al. [27] | 2007 | USA | AMI, HF, PN | To investigate if participation in public reporting and pay for performance accelerate improvements in hospital care. |
| O’Brien et al. [140] | 2007 | USA | Adult cardiac surgery | To describe statistical considerations relevant to combining multiple measures into a composite score and then using them to rate providers. |
| Holmboe et al. [108] | 2008 | USA | Diabetes | To investigate the association between physicians’ cognitive skill scores and delivery of process of care. |
| Scholle et al. [142] | 2008 | USA | Chronic care, Acute care | To investigate the reliability of composite indicators to assess physician performance. |
| Jacobson et al. [22] | 2008 | USA | Cancer care | To investigate improvement in oncology practice performance through voluntary participation in the Quality Oncology Practice Initiative. |
| Lewis et al. [21] | 2008 | USA | AMI | To evaluate whether participation in GWTG is associated with greater adherence to guidelines for coronary artery disease. |
| Mehta et al. [112] | 2008 | USA | AMI | To evaluate the degree to which hospital process performance are altered after accounting for hospitals' patient demographics, clinical characteristics, and treatment opportunities. |
| Normand et al. [157] | 2008 | USA | AMI, CHF, PN | To compare identification of superior hospitals for providing financial rewards using 2 different scoring systems. |
| Sequist et al. [82] | 2008 | USA | Disease management | To examine association between clinical performance and patient experiences. |
| Shwartz et al. [141] | 2008 | USA | AMI, HF, PN | To compare two different approaches for calculating a composite score. |
| Sperl-Hillen et al. [23] | 2008 | USA | Diabetes | To evaluate the effect of an advanced access program on quality of diabetes care. |
| Bilimoria et al. [143] | 2009 | USA | Melanoma | To develop quality indicators for melanoma and to assess hospital-level adherence with these measures in the US. |
| Brush et al. [42] | 2009 | USA | AMI, HF | To investigate if participation in a state-wide collaborative initiative improved the quality of care for patients with AMI and HF. |
| Colwell et al. [113] | 2009 | USA | Pre-hospital care for patients with chest pain | To determine how well paramedics in an urban public hospital system deliver high-quality, comprehensive care for patients with nontraumatic chest pain. |
| Glickman et al. [12] | 2009 | USA | AMI, HF | To develop a process measure adherence score and to compare associations of hospital adherence to this score and adherence to a composite score based on the CMS scoring system with mortality. |
| Halim et al. [129] | 2009 | USA | Troponin elevation | To explore relationships between sex and treatment and outcomes among patients with troponin 1 to 1.5× ULN. |
| Holbrook et al. [18] | 2009 | Canada | Diabetes | To investigate whether electronic decision support encourages timely interventions and improve the management of diabetes in community-based primary care. |
| Kaplan et al. [138] | 2009 | USA | Diabetes | To estimate the physician effect on quality and creating composite measures. |
| López et al. [114] | 2009 | USA | AMI, HF, PN | To investigate link between having hospitalists and performance on hospital-level quality indicators. |
| Schwamm et al. [41] | 2009 | USA | Stroke | To establish whether participation in Get with the Guidelines-Stroke was associated with improvements in adherence. |
| Arora et al. [83] | 2010 | USA | Functional decline | To assess the relationship between process quality of care for hospitalized elders and functional decline. |
| Blustein et al. [115] | 2010 | USA | AMI, HF | To investigate the association between hospital performance and local economic and human resources. |
| Birtcher et al. [46] | 2010 | USA | CAD | To determine whether the performance achievement program for Get with The Guidelines-CAD was associated with adherence to guidelines for AMI. |
| Bouadma et al. [19] | 2010 | France | PN | To determine the effect of a multifaceted program aimed at preventing ventilator-acquired pneumonia on compliance with preventive measures. |
| Hicks et al. [43] | 2010 | USA | Asthma, Diabetes, Hypertension | To examine whether health disparities collaboratives reduced disparities in quality by race/ethnicity or insurance status in community health centers nationally. |
| Holmboe et al. [145] | 2010 | USA | Diabetes, HF, Osteoarthritis, Upper respiratory infection, Urinary tract infection, Back pain, Depression | To investigate the feasibility, reliability, and validity of comprehensively assessing physician-level performance in ambulatory practice. |
| Jung et al. [38] | 2010 | USA | Chronic care, Maternity care | To examine the impact of voluntary information disclosure on quality of care in Health Maintenance Organization markets. |
| Kilbourne et al. [146] | 2010 | USA | Bipolar disorder | To implement composite quality metrics for bipolar disorder. |
| Laskey et al. [45] | 2010 | USA | ACS | To investigate the effect of hospital participation in a performance improvement program on regional variation in quality of care and outcomes for ACS. |
| Patterson et al. [84] | 2010 | USA | HF | To examine the relationship between adherence to process measures and mortality and readmission. |
| Reeves et al. [116] | 2010 | USA | Stroke | To investigate patient-level and hospital-level determinants of the quality of acute stroke care. |
| Shafi et al. [85] | 2010 | USA | Trauma | To investigate the correlation between compliance with CMS quality indicators and mortality rates. |
| Stulberg et al. [86] | 2010 | USA | SCIP | To examine the relationship between SCIP process-of-care measures and postoperative infection rates. |
| Willis et al. [144] | 2010 | Australia and UK | Trauma | To investigate the association between hospital level composite index methodologies and mortality. |
| Xian et al. [44] | 2010 | USA | AMI | To establish whether benefits from participation in GWTG-CAD were sustained over time. |
| Baker et al. [30] | 2011 | USA | CAD, HF | To investigate whether providing pre-visit paper quality reminders could improve performance. |
| Couralet et al. [147] | 2011 | France | AMI | To determine the impact on hospital ranking of different aggregation methods when creating a composite score. |
| Eapen et al. [149] | 2011 | USA | AMI | To conduct an observational analysis to determine the influence of the opportunity-based and all-or-none composite measures on hospital rankings. |
| Kapoor et al. [119] | 2011 | USA | HF | To investigate whether comorbid diabetes is associated with quality of care and in-hospital mortality. |
| Morgan et al. [48] | 2011 | USA | AMI, HF, PN, SCIP | To evaluate the impact of Contact Isolation on compliance with process of care quality measures. |
| O’Connor et al. [117] | 2011 | USA | HF | To investigate patient and practice factors associated with improvement in use of guideline-recommended therapies for outpatients with heart failure. |
| Ross et al. [118] | 2011 | United States and Puerto Rico | Stroke | To investigate the correlation of inpatient and outpatient measures of stroke care quality. |
| Shubrook et al. [47] | 2011 | USA | Diabetes | To compare program performance (AOA-CAP) in processes of care and intermediate outcomes. |
| Wang et al. [158] | 2011 | USA | AMI, HF | To examine the degree to which hospital performance for acute myocardial infarction (AMI) and heart failure (HF) care processes are correlated. |
| Zurovac et al. [20] | 2011 | Kenya | Pediatric malaria | To assess whether text-message reminders could improve and maintain adherence to treatment guidelines for outpatient pediatric malaria in Kenya. |
| Ashby et al. [91] | 2012 | USA | Overall | To investigate the relationship of hospital quality and cost per case in Hawaii. |
| Bulger et al. [120] | 2012 | USA | Diabetes | To evaluate the delivery of diabetes care (processes and outcomes) associated with racial categories. |
| De Wet et al. [151] | 2012 | UK | COPD | To analyze the effect of an advanced access program on quality of diabetes care. |
| Flotta et al. [74] | 2012 | Italy | AMI, HF, PN, SCIP | To estimate the uptake to quality indicators that reflect the current evidence-based guidelines. |
| Gale et al. [121] | 2012 | UK | AMI | To investigate age-dependent inequalities in improvements in mortality occur after AMI. |
| Hess et al. [103] | 2012 | USA | Diabetes | To examine the association between physicians' cognitive skills and their performance on a composite measure of diabetes care. |
| Kolfschoten et al. [150] | 2012 | Netherlands | Colon cancer, Rectal cancer | To determine if composite measures based on process indicators are consistent with short-term outcome indicators in surgical colorectal cancer care. |
| Martirosyan et al. [148] | 2012 | Netherlands | Diabetes | To identify the relevant prescribing quality domains of diabetes care as a basis for the selection of a minimal set of prescribing quality indicators. |
| Saleh et al. [90] | 2012 | USA | PN | To examine the association between performance on the Hospital Quality Alliance's pneumonia measures and costs associated with pneumonia discharges. |
| Sequist et al. [89] | 2012 | USA | Chronic care | To assess the relationship between clinical care metrics and patient experiences of care among patients with chronic disease. |
| Sills et al. [88] | 2012 | USA | Asthma | To investigate the association between process and outcome measures of the quality of acute asthma care. |
| Aaronson et al. [87] | 2013 | USA | AMI, HF | To identify an approach to summarizing hospital performance data for AMI or HF that best predicts hospital mortality rates. |
| Appari et al. [49] | 2013 | USA | AMI, HF, PN, SCIP | To estimate the effects of transitions in electronic health record system capabilities on hospital process quality. |
| Simms et al. [152] | 2013 | UK | AMI | To investigate whether a hospital-specific opportunity-based composite score was associated with mortality. |
| Hasegawa et al. [92] | 2013 | Japan | Asthma | To investigate concordance of acute asthma management in emergency departments with recommendations in guidelines and whether guideline concordance was associated with risk of hospital admission. |
| Ji et al. [128] | 2013 | China | Stroke | To examine the association between gross regional product per capita and prehospital management, in-hospital quality of care and functional outcome. |
| McHugh et al. [51] | 2013 | USA | AMI, PN, HF, SCIP | To describe hospital reporting on emergency department related program measures and variation in performance on the ED measures across hospital characteristics. |
| Peacock et al. [53] | 2013 | USA | AMI | To describe the association between SCPC accreditation and hospital quality metric performance. |
| Schiele et al. [122] | 2013 | France | AMI | To quantify the relationship between volume and quality indicators in survivors after AMI. |
| Simms et al. [11] | 2013 | UK | AMI | To investigate the influence of three aggregation methods for an AMI indicator on mortality and hospital rank. |
| Vichare et al. [31] | 2013 | USA | Cancer care | To determine baseline performance rates and practice variation. |
| Kontos et al. [93] | 2014 | USA | AMI | To determine whether hospital-level adherence with process performance measures was associated with in-hospital mortality. |
| Mitchell et al. [54] | 2014 | USA | PN | To determine whether there is an association between clinical decision support system use and quality disparities in pneumonia process indicators between rural and urban hospitals. |
| Paustian et al. [52] | 2014 | USA | Overall | To examine the associations between partial and incremental implementation of the Patient Centered Medical Home model and measures of quality of care. |
| Perlin et al. [50] | 2014 | USA | AMI, HF, PN, SCIP | To investigate the association between implementation of the HCA “Getting to Green” program and quality of care. |
| Ukawa et al. [123] | 2014 | Japan | AMI | To elucidate the hospital characteristics associated with hospital performance and time trends in acute myocardial infarction care. |
| Amoah et al. [154] | 2015 | USA | Primary care | To develop composite quality measures for EHR-enabled primary care practices in NYC. |
| Bogh et al. [57] | 2015 | Denmark | Stroke, HF, Ulcer | To examine whether performance measures improve more in accredited hospitals than in non-accredited hospitals. |
| Dusheiko et al. [94] | 2015 | UK | Overall (somatic and mental health care) | To investigate whether better management of chronic conditions by family practices reduces mortality risk. |
| Herrera et al. [56] | 2015 | Mexico | Stroke | To determine if the implementation of a stroke registry is associated with an improved adherence to the performance measures. |
| Lytle et al. [40] | 2015 | USA | AMI | To investigate the optimum type of data feedback to support quality improvement. |
| Nkoy et al. [58] | 2015 | USA | Asthma | To assess the impact of an evidence-based care process model 5 years after implementation at a tertiary care facility, and its dissemination to 7 community hospitals. |
| Nuti et al. [73] | 2015 | USA | AMI, HF, PN | To determine how the distribution of performance across hospitals has changed. |
| Samuel et al. [136] | 2015 | USA | Colorectal, lung and prostate cancer | To compare multiple approaches for generating cancer care composite measures and evaluate how well composite measures summarize dimensions of cancer care and predict survival. |
| Weng et al. [153] | 2015 | USA | Osteoporosis | To develop a credible clinical performance assessment to measure quality of osteoporosis care and determine performance standards for competent and excellent care. |
| Bosko et al. [81] | 2016 | USA | Chronic care | To assess the relationship between patient satisfaction and clinical quality. |
| Hsieh et al. [17] | 2016 | Taiwan | Stroke | To improve stroke care quality via a collaborative model, the Breakthrough Series-Stroke activity, in a nationwide, multi-center activity in Taiwan. |
| Kinnier et al. [155] | 2016 | USA | Surgery | To develop a novel composite process measure for venous thromboembolism prophylaxis. |
| Li et al. [60] | 2016 | China | Stroke | To determine whether adherence to quality metrics had improved after stroke quality management initiatives are implemented. |
| Mitchell et al. [55] | 2016 | USA | PN | To examine the association between clinical decision support system use and the treatment of pneumonia care within high-minority and low-minority areas. |
| Pan et al. [126] | 2016 | China | Stroke | To investigate the association between socioeconomic status and quality of stroke care. |
| Policardo et al. [125] | 2016 | Italy | Diabetes | To investigate whether surgery due to newly diagnosed cancer may modify quality of diabetes' management. |
| Seghieri et al. [124] | 2016 | Italy | Diabetes | To evaluate whether adherence to process quality-of-care-indicator in diabetes, is able to predict, equally in men and women, hospitalization or mortality risk. |
| Aliprandi-Costa et al. [156] | 2017 | Australia | ACS | To investigate the contribution of the composite of process indicators as a measure of hospital performance in the management of ACS. |
| Baack Kukreja et al. [59] | 2017 | USA | Bladder cancer | To determine if patients managed with a cystectomy enhanced recovery pathway have improved quality of care. |
| Chui et al. [101] | 2017 | USA | PCI | To investigate association between PCI process and outcome measures. |
| Cross et al. [133] | 2017 | USA | Overall | To investigate what types of practices perform best for high-needs patients. |
| Dentan et al. [67] | 2017 | France | Prescription appropriateness | To assess the appropriateness of linezolid use in French hospitals. |
| Diop et al. [61] | 2017 | Canada | Diabetes, HF, COPD | To investigate whether enrolment in multidisciplinary team-based primary care practice improve adherence to guideline-recommended processes of care. |
| Falstie-Jensen et al. [65] | 2017 | Denmark | Stroke, Diabetes, COPD, HF, Hip fracture, Ulcer | To examine the association between compliance with accreditation and recommended hospital care. |
| Loftus et al. [29] | 2017 | USA | Bowel surgery | To evaluate the association between process of care indicators and outcomes. |
| Mason et al. [100] | 2017 | USA | Colon cancer | To evaluate the impact of care at high-performing hospitals on the National Quality Forum colon cancer metrics. |
| McDermott et al. [132] | 2017 | USA | Stroke | To investigate sex disparities in stroke quality of care. |
| Ndumele et al. [63] | 2017 | USA | Chronic care, Maternal care | To investigate the association between health plan exit from Medicaid managed care and quality of care. |
| Peterson et al. [64] | 2017 | USA | Diabetes | To investigate the association between extending CareFirst's medical home program and quality of care, utilization, and spending. |
| Plackett et al. [76] | 2017 | USA | Trauma | To investigate whether guidelines are being followed in military operations. |
| Ryan et al. [62] | 2017 | USA | AMI, HF, PN | To evaluate whether quality improved more in acute care hospitals that were exposed to Hospital Value-Based Purchasing programs than in control hospitals. |
| Schneider et al. [34] | 2017 | USA | SCIP | To measure adherence to the evidence-based practices before and after the quality improvement campaign. |
| Smith et al. [99] | 2017 | USA | Overall | To examine the effects of provider characteristics on home health agency performance on patient experience of care and process measures. |
| Su et al. [131] | 2017 | China | Breast cancer | To examine the association between different proportions of reimbursement and quality of recommended breast cancer care and length of hospital stay. |
| Alvarez Morán et al. [28] | 2018 | Mali | Severe acute malnutrition | To assess the quality of care delivered by healthcare workers. |
| Hong et al. [72] | 2018 | Korea | PN | To evaluate the quality of care among hospitalized patients with community-acquired pneumonia in Korea. |
| Ido et al. [98] | 2018 | USA | Stroke | To investigate the relation between quality of care and its impact on one-year mortality. |
| Spece et al. [96] | 2018 | USA | COPD | To examine the quality of inpatient COPD care and the associations with readmission and mortality. |
| Starks et al. [66] | 2018 | USA | In-hospital cardiac arrest | To examine if Get with The Guidelines-Resuscitation (GWTG-R) participation duration was associated with improved care processes. |
| Wang et al. [16] | 2018 | China | Stroke | To determine whether a multifaceted quality improvement intervention can improve adherence to evidence-based performance measures. |
| Zhang et al. [79] | 2018 | China | Stroke | To evaluate the correlations between hospital performance measures and outcomes among patients with acute ischemic stroke. |
| Barbayannis et al. [97] | 2019 | USA | AMI | To assess the relation between state-wide hospital performance reports on AMI and cardiovascular outcomes. |
| Bintabara et al. [71] | 2019 | Tanzania | Antenatal care | To assess provider adherence to first-visit antenatal care standards and to apply stratified analysis to identify associated factors in Tanzania. |
| Cadilhac et al. [32] | 2019 | Australia | Stroke | To determine the impact of a multicomponent program involving financial incentives and quality improvement interventions, on stroke care processes. |
| Congiusta et al. [95] | 2019 | USA | Overall | To investigate the relation between clinical quality and patient experience in the adult ambulatory setting. |
| Desai et al. [127] | 2019 | USA | AMI | To examine trends in patient- and hospital-level performance, identify disparities in the performance across sociodemographic groups. |
| Kovács et al. [106] | 2019 | Hungary | Primary care | To investigate the association between gender of the general practitioner and the quality of primary care in Hungary and to assess the size of the gender impact. |
| Rehman et al. [80] | 2019 | Pakistan | AMI | To evaluate the quality of process care and its impact on in-hospital outcomes among AMI patients in Pakistan. |
| Seghieri et al. [130] | 2019 | Italy | Diabetes | To investigate whether migrants are correctly addressed to a standard quality of care for diabetes and are properly followed up. |
| Wang et al. [102] | 2019 | China | Breast cancer | To explore key indicators that can improve the quality of care and factors that may affect the use of these indicators. |
| Wu et al. [39] | 2019 | China | ACS | To determine whether a multifaceted quality of care improvement intervention could improve clinical outcomes among patients with ACS. |
| Getachew et al. [78] | 2020 | Ethiopia | Sick child services | To examine structural and process quality of services for sick children and its association with client satisfaction at health facilities in Ethiopia. |
| Heselmans et al. [35] | 2020 | Belgium | Diabetes | To assess the effectiveness of EBMeDS use in improving diabetes care. |
| Levine et al. [104] | 2020 | USA | AMI, Stroke | To explore the influence of mild cognitive impairment on physician decision-making and recommendations for effective treatments for stroke and AMI. |
| Katzenellenbogen et al. [68] | 2020 | Australia | Acute rheumatic fever and rheumatic heart disease | To investigate the delivery of services for the management of ARF and RHD in primary healthcare centers participating in the Audit and Best Practice for Chronic Disease National Research Partnership project. |
| Murtas et al. [134] | 2020 | Italy | Colorectal cancer | To evaluate the adherence of clinical pathways to clinical guidelines provided at the hospital level, for colorectal cancer care. |
| Al Qawasmeh et al. [105] | 2020 | Jordan | Stroke | To assess the degree of physician adherence to guidelines and the patient specific factors that affect their prescribing patterns. |
| Ranasinghe et al. [69] | 2020 | Sri Lanka | AMI | To evaluate the quality of care for patients admitted with acute myocardial infarction in a tertiary hospital in Colombo. |
| Schumacher et al. [37] | 2020 | USA | Pediatric, Emergency care | To describe how often residents complete resident-sensitive quality measures, when they are implemented in the clinical environment. |
| Starks et al. [77] | 2020 | USA | In-hospital cardiac arrest | To examine processes of care and outcomes of in-hospital cardiac arrest for patients on maintenance dialysis compared with non-dialysis patients. |
| Tawfiq et al. [33] | 2020 | Afghanistan | Pediatric, Primary care | To investigate the effects of training courses in integrated management of childhood illness (IMCI) on quality of care in public primary healthcare facilities in Afghanistan. |
| Wang et al. [139] | 2020 | China | Stroke | To develop a tool for identifying performance-based outlier hospitals based on risk-adjusted adherence rates of process indicators. |
| Wang et al. [70] | 2020 | China | Stroke | To assess the prevalence and trends in community-based risk factors, clinical characteristics, management, and outcomes of patients hospitalized with stroke in China. |
| Wolfe et al. [36] | 2020 | USA | Pediatric, in-hospital cardiac arrest | To the association between DEVs and survival outcomes, evaluate the association between composite measure DEV with survival outcomes and evaluate the association between composite measure and Airway Dev on survival. |

ACS = Acute coronary syndromes, AMI = Acute myocardial infarction, CAD = Coronary Artery Disease, COPD = Chronic obstructive pulmonary disease, HF = Heart failure, PCI = Percutaneous coronary intervention, PN = Pneumonia, SCIP = Surgical care and infection prevention
