# Supplementary material for: Composite measures of quality of health care: Evidence mapping of methodology and reporting
Source: PLoS One. 2022 May 12;17(5):e0268320. doi: 10.1371/journal.pone.0268320 (PMC9098058; doi:10.1371/journal.pone.0268320)
Supplement: S2 Table — (DOCX) [file pone.0268320.s004.docx]

**S2 Table. Context, methods and methodological considerations in the included publications**

| Publication | Method | Main purpose* | Provides any limitation | Provides any justification | Provides any advantage | Mentions alternatives |
| --- | --- | --- | --- | --- | --- | --- |
| Williams et al. 2006 [75] | Overall percentage, Equal weights | Operational, Group 2 | + | + | + | + |
| Landon et al. 2006 [111] | Overall percentage, Equal weights | Research, Group 4 | - | - | - | - |
| Lewis et al. 2008 [21] | Overall percentage, Equal weights | Operational, Group 1 | - | - | - | - |
| Scholle et al. 2008 [142] | Overall percentage, Equal weights | Research, Group 5 | + | + | + | + |
| Mehta et al. 2008 [112] | Overall percentage, Equal weights | Research, Group 4 | - | - | - | - |
| Sequist et al. 2008 [82] | Overall percentage, Equal weights | Research, Group 3 | - | - | - | - |
| Schwamm et al. 2009 [41] | Overall percentage, Equal weights | Operational, Group 1 | - | - | - | - |
| López et al. 2009 [114] | Overall percentage, Equal weights | Research, Group 4 | - | - | - | - |
| Xian et al. 2010 [44] | Overall percentage, Equal weights | Operational, Group 2 | - | - | - | - |
| Shafi et al. 2010 [85] | Overall percentage, Equal weights | Research, Group 3 | - | - | - | - |
| Blustein et al. 2010 [115] | Overall percentage, Equal weights | Research, Group 4 | - | - | - | - |
| Baker et al. 2011 [30] | Overall percentage, Equal weights | Operational, Group 1 | - | - | - | - |
| Wang et al. 2011 [158] | Overall percentage, Equal weights | Research, Group 6 | - | - | - | - |
| Saleh et al. 2012 [90] | Overall percentage, Equal weights | Research, Group 3 | - | - | - | - |
| Hess et al. 2012 [103] | Overall percentage, Expert weights | Research, Group 4 | - | - | - | - |
| Appari et al. 2013 [49] | Overall percentage, Equal weights | Operational, Group 1 | - | - | - | - |
| Peacock et al. 2013 [53] | Overall percentage, Equal weights | Operational, Group 1 | + | - | + | - |
| McHugh et al. 2013 [51] | Overall percentage, Equal weights | Operational, Group 1 | - | - | - | - |
| Aaronson et al. 2013 [87] | Overall percentage, Equal weights | Research, Group 3 | - | + | + | + |
| Simms et al. 2013 [152] | Overall percentage, Equal weights | Research, Group 5 | + | + | + | + |
| Mitchell et al. 2014 [54] | Overall percentage, Equal weights | Operational, Group 1 | - | + | - | - |
| Perlin et al. 2014 [50] | Overall percentage, Equal weights | Operational, Group 1 | - | - | - | - |
| Paustian et al. 2014 [52] | Overall percentage, Equal weights | Operational, Group 1 | - | - | + | - |
| Kontos et al. 2014 [93] | Overall percentage, Equal weights | Research, Group 3 | - | - | - | + |
| Ukawa et al. 2014 [123] | Overall percentage, Equal weights | Research, Group 4 | - | + | + | + |
| Nuti et al. 2015 [73] | Overall percentage, Equal weights | Operational Group 2 | - | - | - | - |
| Herrera et al. 2015 [56] | Overall percentage, Equal weights | Operational, Group 1 | - | - | - | - |
| Lytle et al. 2015 [40] | Overall percentage, Equal weights | Operational, Group 1 | - | - | - | - |
| Mitchell et al. 2016 [55] | Overall percentage, Equal weights | Operational, Group 1 | - | + | + | - |
| Baack Kukreja et al. 2016 [59] | Overall percentage, Equal weights | Operational, Group 1 | - | - | + | - |
| Li et al. 2016 [60] | Overall percentage, Equal weights | Operational, Group 1 | - | - | - | - |
| Hsieh et al. 2016 [17] | Overall percentage, Equal weights | Operational, Group 1 | - | - | - | - |
| Bosko et al. 2016 [81] | Overall percentage, Equal weights | Research, Group 3 | - | - | - | - |
| Loftus et al. 2017 [29] | Overall percentage, Equal weights | Operational, Group 1 | - | - | - | - |
| Plackett et al. 2017 [76] | Overall percentage, Equal weights | Operational Group 2 | - | - | - | - |
| Chui et al. 2017 [101] | Overall percentage, Equal weights | Research, Group 3 | - | - | + | - |
| Starks et al. 2018 [66] | Overall percentage, Equal weights | Operational Group 1 | - | - | + | - |
| Zhang et al. 2018 [79] | Overall percentage, Equal weights | Research, Group 3 | - | - | - | - |
| Cadilhac et al. 2019 [32] | Overall percentage, Equal weights | Operational, Group 1 | - | - | - | - |
| Kovács et al. 2019 [106] | Overall percentage, Equal weights | Research, Group 4 | - | - | - | - |
| Tawfiq et al. 2020 [33] | Overall percentage, Equal weights | Operational Group 1 | - | - | - | - |
| Heselmans et al. 2020 [35] | Overall percentage, Equal weights | Operational, Group 1 | - | - | - | - |
| Ranasinghe et al. 2020 [69] | Overall percentage, Equal weights | Operational, Group 2 | - | - | - | - |
| Starks et al. 2020 [77] | Overall percentage, Equal weights | Research, Group 3 | - | - | - | - |
| Levine et al. 2020 [104] | Overall percentage, Equal weights | Research, Group 4 | - | - | - | - |
| Min et al. 2005 [107] | Patient average, Equal weights | Research, Group 4 | - | - | - | - |
| Halterman et al. 2006 [135] | Patient average, Expert weights | Research, Group 5 | + | + | + | - |
| Glickman et al. 2007 [24] | Patient average, Equal weights | Operational, Group 1 | - | - | - | - |
| Landon et al. 2007 [26] | Patient average, Equal weights | Operational, Group 1 | - | - | - | - |
| Holbrook et al. 2009 [18] | Patient average, Unequal weights | Operational, Group 1 | - | - | - | - |
| Arora et al. 2009 [83] | Patient average, Equal weights | Research, Group 3 | - | - | + | - |
| Halim et al. 2009 [129] | Patient average, Equal weights | Research, Group 4 | - | - | - | - |
| Kaplan et al. 2009 [138] | Patient average, Equal weights | Research, Group 5 | - | - | + | - |
| Hicks et al. 2010 [43] | Patient average, Equal weights | Operational, Group 1 | - | - | - | - |
| Reeves et al. 2010 [116] | Patient average, Equal weights | Research, Group 4 | + | - | + | - |
| Flotta et al. 2012 [74] | Patient average, Equal weights | Operational, Group 2 | - | - | - | - |
| Gale et al. 2012 [121] | Patient average, Equal weights | Research, Group 4 | - | - | - | - |
| Ji et al. 2013 [128] | Patient average, Equal weights | Research, Group 4 | - | - | - | - |
| Hasegawa et al. 2013 [92] | Patient average, Equal weights | Research, Group 3 | - | - | - | - |
| Nkoy et al. 2015 [58] | Patient average, Equal weights | Operational, Group 1 | - | - | - | - |
| Pan et al. 2016 [126] | Patient average, Equal weights | Research, Group 4 | - | - | - | - |
| Cross et al. 2017 [133] | Patient average, Equal weights | Research, Group 4 | - | - | - | - |
| Bintabara et al. 2019 [71] | Patient average, Equal weights | Operational, Group 2 | - | - | - | - |
| Wu et al. 2019 [39] | Patient average, Equal weights | Operational, Group 1 | - | - | - | - |
| Wang et al. 2019 [102] | Patient average, Equal weights | Research, Group 4 | - | - | - | - |
| Rehman et al. 2019 [80] | Patient average, Equal weights | Research, Group 3 | + | - | + | - |
| Schumacher et al. 2020 [37] | Patient average, Equal weights | Operational, Group 1 | - | - | - | - |
| Katzenellenbogen et al. 2020 [68] | Patient average, Equal weights | Operational, Group 2 | - | - | - | - |
| Correa-de-Araujo et al. 2006 [109] | All-or-none scoring | Research, Group 4 | - | - | - | - |
| Lindenauer et al. 2006 [110] | All-or-none scoring | Research, Group 4 | - | - | - | - |
| Krantz et al. 2007 [25] | All-or-none scoring | Operational, Group 1 | - | + | - | - |
| Sperl-Hillen et al. 2008 [23] | All-or-none scoring | Operational, Group 1 | - | + | + | - |
| Holmboe et al. 2008 [108] | All-or-none scoring | Research, Group 4 | - | - | - | - |
| Brush et al. 2009 [42] | All-or-none scoring | Operational, Group 1 | - | - | - | - |
| Colwell et al. 2009 [113] | All-or-none scoring | Research, Group 4 | - | - | - | - |
| Stulberg et al. 2010 [86] | All-or-none scoring | Research, Group 3 | - | + | + | - |
| Kilbourne et al. 2010 [146] | All-or-none scoring | Research, Group 5 | + | + | + | + |
| Shubrook et al. 2011 [47] | All-or-none scoring | Operational, Group 1 | - | - | - | - |
| Ross et al. 2011 [118] | All-or-none scoring | Research, Group 4 | - | + | - | - |
| Bulger et al. 2012 [120] | All-or-none scoring | Research, Group 4 | - | - | - | - |
| De Wet et al. 2012 [151] | All-or-none scoring | Research, Group 5 | + | + | + | - |
| Vichare et al. 2013 [31] | All-or-none scoring | Operational, Group 1 | - | - | + | - |
| Schiele et al. 2013 [122] | All-or-none scoring | Research, Group 4 | + | + | + | + |
| Kinnier et al. 2016 [155] | All-or-none scoring | Research, Group 5 | - | - | + | - |
| Schneider et al. 2017 [34] | All-or-none scoring | Operational, Group 1 | - | + | - | - |
| Dentan et al. 2017 [67] | All-or-none scoring | Operational Group 2 | - | - | - | - |
| Falstie-Jensen et al. 2017 [65] | All-or-none scoring | Operational, Group 1 | - | - | - | - |
| Peterson et al. 2017 [64] | All-or-none scoring | Operational, Group 1 | - | - | - | - |
| Aliprandi-Costa et al. 2017 [156] | All-or-none scoring | Research, Group 5 | - | + | + | + |
| Su et al. 2017 [131] | All-or-none scoring | Research, Group 4 | - | - | - | - |
| Alvarez Morán et al. 2018 [28] | All-or-none scoring | Operational, Group 1 | - | - | - | - |
| Spece et al. 2018 [96] | All-or-none scoring | Research, Group 3 | - | - | - | - |
| Desai et al. 2019 [127] | All-or-none scoring | Research, Group 4 | - | + | + | - |
| Wolfe et al. 2020 [36] | All-or-none scoring | Operational, Group 1 | - | - | - | - |
| Al Qawasmeh et al. 2020 [105] | All-or-none scoring | Research, Group 4 | - | - | - | - |
| Seghieri et al. 2016 [124] | Threshold approach | Research, Group 4 | - | - | - | - |
| Policardo et al. 2016 [125] | Threshold approach | Research, Group 4 | - | - | - | - |
| Seghieri et al. 2019 [130] | Threshold approach | Research, Group 4 | - | - | - | - |
| Halasyamani et al. 2007 [137] | Indicator average, Equal weights | Research, Group 5 | - | - | - | - |
| Jung et al. 2010 [38] | Indicator average, Equal weights | Operational Group 1 | - | - | + | - |
| Bouadma et al. 2010 [19] | Indicator average, Equal weights | Operational, Group 1 | - | - | - | - |
| Holmboe et al. 2010 [145] | Indicator average, Equal weights | Research, Group 5 | + | + | + | - |
| Sequist et al. 2012 [89] | Indicator average, Equal weights | Research, Group 3 | - | - | - | - |
| Martirosyan et al. 2012 [148] | Indicator average, Equal weights | Research, Group 5 | - | - | + | - |
| Samuel et al. 2015 [136] | Indicator average, Equal weights | Research, Group 5 | - | + | + | - |
| Amoah et al. 2015 [154] | Indicator average, Equal weights | Research, Group 5 | - | - | + | - |
| Dusheiko et al. 2015 [94] | Indicator average, Unequal weights | Research, Group 3 | - | - | - | - |
| Weng et al. 2015 [153] | Indicator average, Expert weights | Research, Group 5 | - | + | + | - |
| Ndumele et al. 2017 [63] | Indicator average, Equal weights | Operational, Group 1 | - | - | - | - |
| Ryan et al. 2017 [62] | Indicator average, Equal weights | Operational Group 1 | - | - | - | - |
| Smith et al. 2017 [99] | Indicator average, Equal weights | Research, Group 4 | + | - | - | - |
| Hong et al. 2018 [72] | Indicator average, Expert weights | Operational, Group 1 | - | - | - | - |
| Wang et al. 2020 [139] | Indicator average, Equal weights | Research, Group 5 | + | - | + | + |
| Bilimoria et al. 2009 [143] | Threshold approach on indicator level, indicators equally weighted | Research, Group 5 | - | + | + | - |
| Jacobson et al. 2008 [22] | Unclear methodology | Operational, Group 1 | - | - | - | - |
| Ashby et al. 2012 [91] | Unclear methodology | Research, Group 3 | - | - | - | - |
| Barbayannis et al. 2019 [97] | Unclear methodology | Research, Group 3 | - | - | - | - |
| Congiusta et al. 2019 [95] | Unclear methodology | Research, Group 3 | - | - | - | - |
| Getachew et al. 2020 [78] | Principal component analysis | Research, Group 3 | - | - | - | - |
| Lindenauer et al. 2007 [27] | Overall percentage with equal weights and all-or-none scoring | Operational, Group 1 | - | + | - | NA |
| Laskey et al. 2010 [45] | Overall percentage with equal weights and all-or-none scoring | Operational, Group 1 | - | - | - | NA |
| Birtcher et al. 2010 [46] | Overall percentage with equal weights and all-or-none scoring | Operational, Group 1 | - | - | - | NA |
| Patterson et al. 2010 [84] | Overall percentage with equal weights and all-or-none scoring | Research Group 3 | - | - | - | NA |
| Eapen et al. 2011 [149] | Overall percentage with equal weights and all-or-none scoring | Research, Group 5 | + | + | + | NA |
| O’Connor et al. 2011 [117] | Overall percentage with equal weights and all-or-none scoring | Research, Group 4 | - | - | - | NA |
| Kapoor et al. 2011 [119] | Overall percentage with equal weights and all-or-none scoring | Research, Group 4 | - | - | - | NA |
| Bogh et al. 2015 [57] | Overall percentage with equal weights and all-or-none scoring | Operational, Group 1 | - | + | - | NA |
| Wang et al. 2020 [70] | Overall percentage with equal weights and all-or-none scoring | Operational, Group 2 | - | - | - | NA |
| Ido et al. 2018 [98] | Patient average (indicators are equally weighted, patients are unequally weighted) and all-or-none scoring | Research, Group 3 | + | + | - | NA |
| Sills et al. 2012 [88] | Patient average with equal weights and all-or-none scoring | Research Group 3 | - | - | - | NA |
| McDermott et al. 2017 [132] | Patient average with equal weights and all-or-none scoring | Research, Group 4 | - | - | - | NA |
| Wang et al. 2018 [16] | Patient average with equal weights and all-or-none scoring | Operational, Group 1 | - | - | - | NA |
| Morgan et al. 2011 [48] | All-or-none scoring and threshold approach | Operational, Group 1 | - | - | - | NA |
| Zurovac et al. 2011 [20] | All-or-none scoring and threshold approach | Operational, Group 1 | - | - | - | NA |
| Diop et al. 2017 [61] | All-or-none scoring and threshold approach | Operational, Group 1 | - | - | - | NA |
| Mason et al. 2017 [100] | All-or-none scoring and threshold approach | Research, Group 3 | - | + | + | NA |
| O’Brien et al. 2007 [140] | Overall percentage with equal weights, Indicator average with equal weights, all-or-none scoring and item response theory | Research, Group 5 | + | + | + | NA |
| Shwartz et al. 2008 [141] | Overall percentage with equal weights, Bayesian hierarchical latent models, Bayesian-estimated weights | Research, Group 5 | + | + | + | NA |
| Normand et al. 2008 [157] | Overall percentage with equal weights and indicator average with item response theory weights | Research, Group 5 | + | + | - | NA |
| Glickman et al. 2009 [12] | Overall percentage with equal weights and indicator average with PCA-based weights | Research, Group 5 | - | + | - | NA |
| Willis et al. 2010 [144] | Overall percentage with equal weights and overall percentage with PCA-based weights | Research, Group 5 | + | + | + | NA |
| Couralet et al. 2011 [147] | Indicator average with equal weights, indicator average with expert weights and benefit of doubt weights, all-or-none scoring and latent variable models | Research, Group 5 | + | + | + | NA |
| Kolfschoten et al. 2012 [150] | Overall percentage with equal weights, patient average with equal weights, all-or-none scoring and threshold approach | Research, Group 5 | - | + | + | NA |
| Simms et al. 2013 [11] | Overall percentage with equal weights, overall percentage with regression weights and all-or-none scoring | Research, Group 5 | + | + | + | NA |
| Murtas et al. 2020 [134] | Overall percentage with equal weights and item response theory | Research, Group 5 | + | + | + | NA |

* Main aim of the study is categorized as, **Group 1:** To investigate the effect of a program participation, implementation or intervention (operational use), **Group 2:** Pure evaluation of quality of care in healthcare providers and/or comparison of healthcare providers (operational use), **Group 3:** To assess the association between process and outcome indicators (research purposes), **Group 4:** To assess the association between hospital and/or patient characteristics and quality of care (research purposes), **Group 5:** To investigate use, implementation or comparison of composite indicators (research purposes), **Group 6:** To assess the correlation between quality of care for different diseases/care areas (research purposes).
