# Supplementary material for: Composite measures of quality of health care: Evidence mapping of methodology and reporting
Source: PLoS One. 2022 May 12;17(5):e0268320. doi: 10.1371/journal.pone.0268320 (PMC9098058; doi:10.1371/journal.pone.0268320)
Supplement: S3 Table — (DOCX) [file pone.0268320.s005.docx]

**S3 Table. Aggregation methods used in publications**

| Method | | Number of papers n=145 | References |
| --- | --- | --- | --- |
| Overall percentage | Publications that “only” used overall percentage | 46 (32%) | 17, 21, 29, 30, 32, 33, 35, 40, 41, 44, 49-56, 59, 60, 66, 69, 73, 75-77, 79, 81, 82, 85, 87, 90, 93, 101, 103, 104, 106, 111, 112, 114, 115, 123, 142, 144, 152, 158 |
|  | Publications that used overall percentage method (includes publications with multiple approaches) | 61 (42%) | 11, 17, 21, 27, 29, 30, 32, 33, 35, 40, 41, 44-46, 49-57, 59, 60, 66, 69, 70, 73, 75-77, 79, 81, 82, 84, 85, 87, 90, 93, 101, 103, 104, 106, 111, 112, 114, 115, 117, 119, 123, 134, 140-142, 144, 149, 150, 152, 157, 158 |
| Patient average | Publications that “only” used patient average | 23 (16%) | 18, 24, 26, 37, 39, 43, 58, 68, 71, 74, 80, 83, 92, 102, 107, 116, 121, 126, 128, 129, 133, 135, 138 |
|  | Publications that used patient average method (includes publications with multiple approaches) | 29 (20%) | 12, 16, 18, 24, 26, 37, 39, 43, 58, 68, 71, 74, 80, 83, 88, 92, 98, 102, 107, 116, 121, 126, 128, 129, 132, 133, 135, 138, 150 |
| All-or-none scoring | Publications that “only” used all-or-none scoring | 27 (19%) | 23, 25, 28, 31, 34, 36, 42, 47, 64, 65, 67, 86, 96, 105, 108-110, 113, 118, 120, 122, 127, 131, 146, 151, 155, 156 |
|  | Publications that used all-or-none scoring method (includes publications with multiple approaches) | 48 (33%) | 11, 16, 20, 23, 25, 27, 28, 31, 34, 36, 42, 45-48, 57, 61, 64, 65, 67, 70, 84, 86, 88, 96, 98, 100, 105, 108-110, 113,117-120, 122, 127, 131, 132, 140, 146, 147, 149-151, 155, 156 |
| Indicator average | Publications that “only” used indicator average | 15 (10%) | 19, 38, 62, 63, 72, 89, 94, 99, 136, 137, 139, 145, 148, 153, 154 |
|  | Publications that used indicator average method (includes publications with multiple approaches) | 19 (13%) | 12, 19, 38, 62, 63, 72, 89, 94, 99, 136, 137, 139, 140, 145, 147, 148, 153, 154, 157 |
| 70% standard and other thresholds | Publications that “only” used 70% standard and other thresholds approach | 3 (2%) | 124, 125, 130 |
|  | Publications that used 70% standard and other thresholds approach (includes publications with multiple approaches) | 8 (6%) | 20, 48, 61, 100, 124, 125, 130, 150] |
| Other approaches | Publications where other approaches were present | 4 (3%) | 78, 141, 143, 147 |
