# Supplementary material for: Composite measures of quality of health care: Evidence mapping of methodology and reporting
Source: PLoS One. 2022 May 12;17(5):e0268320. doi: 10.1371/journal.pone.0268320 (PMC9098058; doi:10.1371/journal.pone.0268320)
Supplement: S4 Table — (DOCX) [file pone.0268320.s006.docx]

**S4 Table. Examples for included indicators for each aggregation method**

| **Method** | **Study title, Authors** | **Disease / care area** | **Included indicators in the composite indicator** |
| --- | --- | --- | --- |
| Overall percentage | **An Organized Approach to Improvement in Guideline Adherence for Acute Myocardial Infarction Results with the Get With The Guidelines Quality Improvement Program**, Lewis et al. | Acute myocardial infarction care | - Aspirin on admission, Aspirin on discharge - β-Blocker on admission - β-Blocker on discharge - ACE inhibitor on discharge - Tobacco cessation counseling - Thrombolytic agent in 30 min - PCI within 120 min |
| Patient average | **Quality of Care for Acute Asthma in Emergency Departments in Japan: A Multicenter Observational Study**, Hasegawa et al. | Acute asthma care | - Inhaled b-agonists in ED - Inhaled anticholinergics in ED - Systemic corticosteroids in ED - Methylxanthines not prescribed in ED - Prescribed oral corticosteroids at discharge - Antibiotics not prescribed in ED - Oral antibiotics not prescribed at discharge - Assessment of PEF |
| Indicator average | **Evaluation of the Quality of Care among Hospitalized Adult Patients with Community-Acquired Pneumonia in Korea**, Hong et al. | Community-acquired pneumonia care | - Oxygenation assessment - Pneumonia severity assessment - Sputum smears - Sputum cultures - Blood cultures prior to first intravenous antibiotics administration, - First dose of antibiotic within 8 hours - Smoking cessation advice /counseling - Screening for pneumococcal vaccination |
| All-or-none scoring | **Correlation of Inpatient and Outpatient Measures of Stroke Care Quality within Veterans Health Administration Hospitals**, Ross et al. | Stroke care | - Antithrombotic therapy - Antilipidemic therapy - Anticoagulation for atrial fibrillation - Tobacco cessation counseling for smokers |
| 70% and other tresholds | **Adherence to diabetes care process indicators in migrants as compared to non-migrants with diabetes: a retrospective cohort study**, Seghieri et al. | Diabetes care | Annual assessment of HbA1c  Eye examination  Serum lipids measurement, Microalbuminuria screening |
| Principal component analysis | **Assessing the quality of care in sick child services at health facilities in Ethiopia**, Getachew et al. | Sick child services | - Number of symptoms checked (6 items; Cough or difficult breathing, diarrhea, fever or body hotness, ear problems, unable to drink or breastfeed, vomiting everything, convulsions) - Physical examination of sick child (16 items; Took child’s temperature by thermometer, felt the child for fever or body hotness, Counted respiration (breaths) for 60 s, Auscultated child (listen to chest with stethoscope) or count pulse, Checked skin turgor for dehydration(e.g., pinch abdominal skin), Checked for pallor by looking at palms, Checked for pallor by looking at conjunctiva, Looked into child’s mouth, Checked for neck stiffness, Looked in child’s ear, Felt behind child’s ear, Undressed child to examine (up to shoulders/down to ankles),Pressed both feet to check for edema, Weighed the child, Plotted weight on growth chart, Checked for enlarged lymph nodes in 2 or more of the following sites: neck, axillae, groin) - Information provided to caregiver (5 items; provide general information about feeding/breastfeeding, advise extra fluids during this sickness, advise continued feeding during sickness, name the illness for the caretaker, describe symptoms requiring immediate return for care) - Provider used visual aids - Provider discussed follow-up visit |
