# Supplementary material for: Composite measures of quality of health care: Evidence mapping of methodology and reporting
Source: PLoS One. 2022 May 12;17(5):e0268320. doi: 10.1371/journal.pone.0268320 (PMC9098058; doi:10.1371/journal.pone.0268320)
Supplement: S5 Table — (DOCX) [file pone.0268320.s007.docx]

**S5 Table. Weighting methods used in publications**

| Weighting approach | Numbers of papers n=145 | References |
| --- | --- | --- |
| Weights were not relevant in the publication (publications that used only all-or-none approach) | 27 (19%) | 23, 25, 28, 31, 34, 36, 42, 47, 64, 65, 67, 86, 96, 105, 108-110, 113, 118, 120, 122, 127, 131, 146, 151, 155, 156 |
| Equal weights | 107 (74%) | 11, 12, 16, 17, 19-21, 24, 26, 27, 29, 30, 32, 33, 35, 37-41, 43-46, 48-63, 66, 68-71, 73-77, 79-85, 87-90, 92, 93, 98-102, 104, 106, 107, 111, 112, 114-117, 119, 121, 123-126, 128-130, 132-134, 136-145, 147-150, 152-154, 157, 158 |
| Expert panel weights | 7 (5%) | 18, 72, 94, 103, 135, 147, 153 |
| Principal component analysis weights | 3 (2%) | 12, 78, 144 |
| Item Response Theory | 3 (2%) | 134, 140, 157 |
| Regression weights | 1 (1%) | 11 |
| Bayesian weights | 1 (1%) | 141 |
| Benefit of doubt | 1 (1%) | 147 |
