# Supplementary material for: Composite measures of quality of health care: Evidence mapping of methodology and reporting
Source: PLoS One. 2022 May 12;17(5):e0268320. doi: 10.1371/journal.pone.0268320 (PMC9098058; doi:10.1371/journal.pone.0268320)
Supplement: S6 Table — (DOCX) [file pone.0268320.s008.docx]

**S6 Table. Justifications for selected methodologies as stated in the included publications**

| **Method** | **Justification** |
| --- | --- |
| **Equal weights** | “Each measure represents a process of care that is recommended by established clinical practice guidelines. The measures were not weighted in anyway, because it is reasonable to expect that all eligible patients should receive care that is consistent with the evidence-based practice guidelines nearly 100% of the time.” [75] |
| **All-or-none** | “Among the different methods of developing composite QIs, we considered the all-or-none method to be most suited to our needs, given that the absence of any one of the QIs included in the composite corresponds to suboptimal management.” [122]  “However the defect-free care composite measure raises the bar for hospitals, requiring that they meet every process measure for which their patients are eligible.” [127]  “After testing all four potential methods for combining measures within domains, the QMTF selected an all/any or none approach. This method is straightforward and intuitive, avoids subjective weighting, sets an appropriately high benchmark for the ideal CABG hospitalization, and performs as well as or better than other methods when applied to actual STS data.” [140]  “All-or-none composite calculated as the percentage of respondents reporting >90% adherence by the hospital to all three new evidence-based components of the SSI reduction bundle. The stringent all-or-none and >90% adherence criteria were selected to measure whether the campaign drove highly-reliable adoption of the entire package of evidence-based practices.” [34]  “Measuring compliance with bundled interventions on a composite ‘all-or-nothing’ basis may provide the healthcare team with a more accurate indicator of care quality and evidence-based care provision [35]. In essence this means every relevant care component should routinely be delivered (or considered) for every single patient on time and every time. Embracing this rationale may act as a greater prompt to improve patient care than the current method of monitoring data with individual bundle elements, which can give a misleading impression of overall performance.” [151]  “Second, we created an “appropriate care measure” by calculating the percentage of patients who received all recommended interventions for a given clinical condition. As compared with composite process measures, appropriate care measures may better represent the interests and likely desires of patients, are more sensitive to subtle improvements, and can help foster a system perspective in quality measurement.^28^” [27] |
| **Opportunity**  **scoring** | “We adopted this method because the numbers of opportunities for each measure were well balanced, which negates the effects of weighting—a common problem associated with this method [20]. Additionally, this method has been used by organizations [22] and previous studies [4,5,7,16].” [123]  “Composite scores based on the 6 pneumonia indicators were calculated using the opportunity method developed by the Hospital Core Performance Measurement Project (HCPM) for the Rhode Island Public Reporting Program for Health Care Services in 1998.^33^ This method was developed to control for individual weighting, missing data, and differences in case volumes” [54]  “This multidimensional approach clearly shows that a composite hospital score, derived from a ‘bundle’ of recommended evidence-based interventions, is a valid and useful indicator of care that is associated with short- and longer-term outcomes. It is also more sophisticated than simple averaging of the individual elements as an implicit weighting is introduced to reflect the number of opportunities present.” [152] |
| **Indicator average** | “We chose to use indicator average method because we intended to assess quality with respect to the processes of care and to avoid potential over representation of frequently triggered individual measures.” [145] |
| **Item Response Theory (IRT)** | “The IRT model is particularly interesting within a decision-making framework. It allows us to distinguish between higher-quality providers by choosing only the procedures with the highest capacity to discriminate, not including indicators with overlapping distribution across providers.” [134] |
| **Threshold on indicator level** | “The individual measures then were assessed at the hospital level, in which institutions were a priori considered to be adherent if 90% or more of patients received care in compliance with the specific quality measure. It is an assumption and premise of the methodology that the measures should be adhered to in nearly all instances. Thus, a 90% threshold was chosen to allow for some variability yet to still require hospitals to achieve a high benchmark. A composite measure of hospital melanoma care was calculated by using the valid measures.” [143] |
| **Patient specific weights** | “A composite measure, taking the proportion of indicated care components a patient received, is an alternative measure of quality of stroke care.^9,10^ However, it does not fully account for the number of indicated care components because delivery of 8 out of 8 would have the same composite measure of 100% as providing 3 out of 3. Thus, the composite measure was weighted by the natural logarithm of the total number of indicated care components. Care requiring 8 elements will have a higher weight [ln(8) = 2.0794] than the one with 3 indicated care components [ln(3) = 1.0986].” [98] |
