# Supplementary material for: Composite measures of quality of health care: Evidence mapping of methodology and reporting
Source: PLoS One. 2022 May 12;17(5):e0268320. doi: 10.1371/journal.pone.0268320 (PMC9098058; doi:10.1371/journal.pone.0268320)
Supplement: S7 Table — (DOCX) [file pone.0268320.s009.docx]

**S7 Table. Examples for methodological considerations counted as “provided”**

| Justification regarding the used methodology | Reference |
| --- | --- |
| “We adopted this method because the numbers of opportunities for each measure were well balanced, which negates the effects of weighting—a common problem associated with this method [20]. Additionally, this method has been used by organizations [22] and previous studies [4, 5, 7, 16].” | 123 |
| “Among the different methods of developing composite QIs, we considered the all-or-none method to be most suited to our needs, given that the absence of any one of the QIs included in the composite corresponds to suboptimal management” | 122 |
| Presented one or more limitation regarding (use of) composite measure(s) | **Reference** |
| “A number of concerns with the use of composite performance indicators have been raised. These include: (1) the potential for loss of detailed performance information; (2) a lack of actionable data to allow targeting of practice; (3) concerns about transparency of composite methodology; and (4) the reliability of hospital ranking.^17,40^ ” | 152 |
| “It is possible that errors could be introduced at any step of assessing the quality of care delivery. The error gets compounded when measures of several quality of care components are aggregated in an all-or-none fashion as in defect-free care.” | 98 |
| Presented one or more advantages regarding (use of) composite measure(s) | **Reference** |
| “Advocates for composite measures note that this approach appropriately assesses and promotes the comprehensive delivery of many components of high-quality care rather than narrowly focusing on individual measures and, therefore, align more closely with patient’s interests.^6,7^ These measures may play an important role with regards to monitoring overall quality of AMI care, identifying opportunities for improvement, and determining whether patterns of care differ across sociodemographic groups.” | 127 |
| “Composites are comprehensive measure sets that provide a more reliable assessment than individual measures alone.^8,9^” | 153 |
| Informed the reader about presence of other approaches to construct composite measures | **Reference** |
| “There are many ways to construct a composite measure. For example, composite scores maybe “compensatory” (a poor score on one individual measure can be compensated by a good score on another measure) or “conjunctive” (all measures must be met to achieve a good score). In addition, individual measures forming a composite score may be transformed and/or weighted differently.” | 87 |
| “Several methods to calculate composite scores have been developed [20,21], and we adopted the opportunity model [22] approach for this study.” | 123 |
